# Association of Diagonal Earlobe Crease (Frank’s Sign) with Intracranial Aneurysm Rupture at Small Sizes

**DOI:** 10.3390/brainsci16080875

**Published:** 2026-08-18

**Authors:** Joel Abraham Velazquez-Castillo, Victor Ramzes Chavez-Herrera, Martin Paredes-Cruz, Pedro Adrian Gonzalez-Zavala, Miguel Adolfo Abdo-Toro, Rabindranath Garcia-Lopez, Ivan Tellez-Medina, Eduardo Ichikawa-Escamilla, Blas Ezequiel Lopez-Felix

**Affiliations:** 1 Department of Neurosurgery, Hospital de Especialidades, Centro Médico Nacional Siglo XXI, Mexico City 06720, Mexico; joelvelazquezc@gmail.com (J.A.V.-C.);; 2 Epidemiology and Health Services Research Unit, Centro Médico Nacional Siglo XXI, Mexico City 06720, Mexico

**Keywords:** intracranial aneurysm, Frank’s sign, cerebrovascular disease, subarachnoid hemorrhage, small aneurysm

## Abstract

Background: The diagonal earlobe crease (DELC), or Frank’s sign, has been associated with systemic vascular degeneration. Whether this cutaneous marker correlates with intracranial aneurysm (IA) instability remains unclear. Methods: We conducted a hospital-based observational registry study including 186 consecutive patients with IA. DELC was dichotomized into absent/low-grade versus high-grade creases. Associations between DELC and aneurysm rupture, rupture at small sizes (<7 mm), and clinical outcome were analyzed using multivariate logistic regression models adjusted for demographic and vascular risk factors. Results: High-grade DELC was significantly associated with overall aneurysm rupture (adjusted OR 3.42, 95% CI 1.39–8.41, *p* = 0.007). Patients with high-grade DELC harbored ruptured aneurysms at significantly smaller mean sizes than those without the sign (6.11 mm vs. 7.94 mm, *p* = 0.015). High-grade DELC was also associated with a distinct anatomical shift toward the anterior communicating artery complex (*p* = 0.021) and was independently associated with rupture at <7 mm (adjusted OR 2.08, 95% CI 1.03–4.21, *p* = 0.041), even after adjustment for aspect ratio and clinical covariates. DELC was not independently associated with post-hemorrhagic clinical outcome. Conclusions: Frank’s sign may represent a readily observable clinical marker associated with IA instability and rupture at conventionally low-risk sizes (<7 mm). These findings support further investigation into DELC as a potential adjunct for individualized risk stratification in patients with small incidental intracranial aneurysms to prevent catastrophic subarachnoid hemorrhage.

## 1. Introduction

Intracranial arterial aneurysms (IAs) represent a major cause of non-traumatic subarachnoid hemorrhage (aSAH), a condition associated with substantial morbidity and mortality worldwide [1]. While treatment paradigms have evolved through cutting-edge imaging and advanced visualization tools, population-wide neurovascular screening (such as Computed Tomography Angiography [CTA] or Magnetic Resonance Angiography [MRA]) remains economically and logistically unfeasible for most healthcare systems [2]. This creates a critical challenge in terms of cost-effectiveness, as widespread imaging yields a high volume of low-risk incidental findings that fail to justify the substantial surveillance costs, leaving a significant proportion of high-risk patients to present only after catastrophic rupture [1]. Consequently, advancing our diagnostic tools requires the identification of non-invasive, accessible markers capable of stratifying aneurysm instability to optimize the allocation of high-cost resources and improve management strategies [2].

The Diagonal Earlobe Crease (DELC), also known as Frank’s sign, was first described in 1973 as a cutaneous marker linked to coronary artery disease [3]. Over the last five decades, several systematic reviews and histopathological studies have validated its association with systemic vascular degeneration, including microvascular loss, elastin fragmentation, and collagen remodeling [4,5]. Notably, these same structural alterations, particularly degradation of the internal elastic lamina and extracellular matrix remodeling, are well recognized as fundamental mechanisms underlying intracranial aneurysm formation and instability [6].

Despite this shared pathophysiology, the potential role of DELC in predicting aneurysm behavior remains largely unexplored in neurosurgery. Previous work from our group first described this association in neurosurgical literature, reporting a high prevalence of DELC in a case series of patients with ruptured IA [7]. Building upon this initial observation, we subsequently conducted the first hospital-based case–control study, which demonstrated that Frank’s sign was an independent predictor of IA presence, with an effect size comparable to that of established vascular risk factors such as hypertension and smoking [8].

Expanding on these results, we hypothesized that this cutaneous marker may be associated with a working hypothesis of increased vascular vulnerability linked to IA rupture at small sizes (<7 mm). The primary objective of this study was therefore to evaluate the independent association between high-grade DELC and aneurysm rupture. Secondary objectives included assessing its relationship with rupture across different aneurysm sizes, particularly rupture at small sizes (<7 mm), and evaluating its impact on post-hemorrhagic clinical outcomes. Under this framework, Frank’s sign represents a readily observable clinical marker bridging systemic connective tissue degeneration with aneurysm wall instability. Ultimately, this framework aligns with current efforts to improve individualized risk stratification and early identification of aneurysm instability before catastrophic rupture occurs through accessible clinical markers.

## 2. Methods

### 2.1. Study Design and Population

A hospital-based observational registry study was conducted using data from an ongoing institutional neurovascular registry at the Hospital de Especialidades, Centro Médico Nacional Siglo XXI, IMSS, Mexico City, Mexico. The present study analyzed a cohort of 186 consecutive patients with confirmed IAs evaluated between June 2021 and January 2026. This work represents the third analytical phase of our departmental research line on Frank’s sign: following our initial pilot series (n = 20) [7] and subsequent case–control study (n = 130 cases vs. 130 controls) [8], the current study analyzed 186 consecutive IA cases. Unlike our previous case–control study [8], non-IA controls were excluded, and the analytical scope was directed internally within the IA cohort to evaluate aneurysm rupture status, anatomical distribution, and small-size rupture dynamics (<7 mm). The study protocol was approved by the local Health Research and Ethics Committee (Registration No. 2024-3601-308).

### 2.2. Clinical Assessment and DELC Evaluation

**Figure 1:**
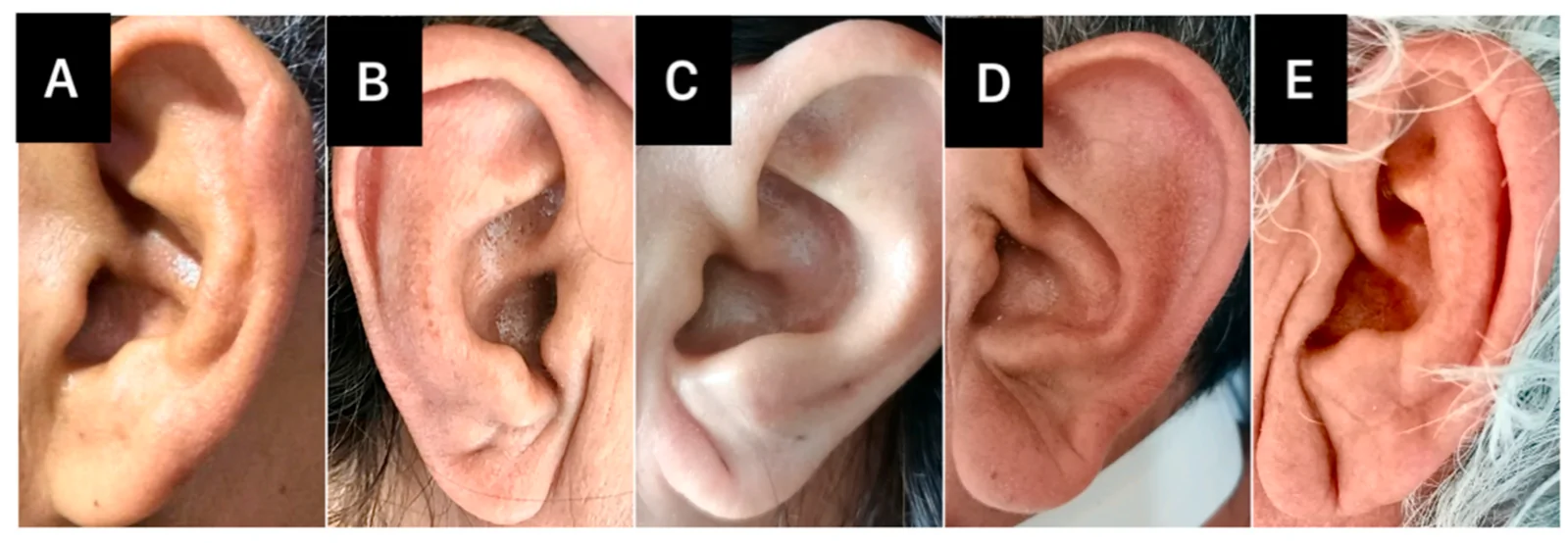
Classification of the diagonal earlobe crease (DELC, Frank’s sign). Representative digital photographs demonstrating the visual grading system used to evaluate DELC severity: (**A**) Type 0: absence of crease; (**B**) Type 1: superficial crease involving <50% of the earlobe; (**C**) Type 2: shallow crease extending across the entire earlobe; (**D**) Type 3: deep crease involving the entire lobe; and (**E**) Type 4: prominent, deeply etched crease extending diagonally across the earlobe. DELC severity was assessed during clinical examination. Reproduced with permission from Chavez-Herrera et al. [8].

Upon admission, demographic characteristics and classical cardiovascular risk factors (including age, sex, smoking status, hypertension, and type 2 diabetes mellitus) were systematically recorded. The presence and grade of Frank’s sign were assessed and photographically documented by two trained physicians blinded to the patients’ neurovascular status. To standardize the assessment, DELC was evaluated using the grading system and interpretation criteria previously validated by our group [8] (Figure 1). In patients exhibiting bilateral asymmetry in earlobe crease severity, the higher DELC grade observed between the two sides was assigned as the definitive patient-level score. For the purposes of statistical analysis, these categories were subsequently dichotomized. Patients with normal earlobes or Type 1–2 creases were classified as Absent/Low-Grade DELC, whereas those with complete creases (Type 3–4) were categorized as High-Grade DELC. To maintain objective evaluation and ensure true observer blinding, earlobe photographs were standardized and cropped strictly to display only the anatomical auricular lobule, removing external clinical cues (such as facial features, endotracheal tubes, or intensive care monitoring gear) prior to evaluation. Inter-observer agreement for visual DELC grading between the two independent evaluators demonstrated strong concordance, as previously validated in our institutional series (Cohen’s Kappa of κ = 0.86) [8].

### 2.3. Aneurysm Characteristics and Severity Grading of aSAH

Radiological evaluation was performed by a neuroradiologist or senior neurosurgeon blinded to the physical examination findings. In patients presenting with aSAH, neurological status at admission was evaluated using the World Federation of Neurosurgical Societies (WFNS) grading system. Hemorrhage severity was assessed on the initial CT scan using the modified Fisher (mFisher) scale. For analytical purposes, clinical presentation was dichotomized into good (WFNS grades I–III) and poor (WFNS grades IV–V) neurological status [9]. Similarly, radiological hemorrhage severity was dichotomized into low (mFisher grades 1–2) and high (mFisher grades 3–4) severity [10]. Aneurysm characteristics were subsequently recorded, including anatomical location (further categorized into anterior and posterior circulation), rupture status (defined by presentation with aSAH), and morphological assessment with aneurysm aspect ratio (AR), calculated according to previously established definitions and maximum dome diameter. All maximum aneurysm dome dimensions and morphological parameters were measured on admission 3D-CTA and independently verified via DSA or intraoperative measurements. For analytical purposes, aneurysm size was dichotomized according to the widely accepted clinical threshold of <7 mm versus ≥7 mm. This cutoff was selected based on landmark natural history studies of unruptured intracranial aneurysms, including the International Study of Unruptured Intracranial Aneurysms (ISUIA) and the UCAS Japan study, which demonstrated a relatively low rupture risk in aneurysms smaller than 7 mm [11,12]. To further evaluate intrinsic vessel wall instability, we specifically analyzed the subgroup of aneurysms that ruptured at small sizes (<7 mm). Finally, clinical state at discharge was assessed using the Glasgow Coma Scale (GCS) to record acute neurological status upon discharge, where GCS 13–15 represented favorable acute recovery and GCS ≤ 12 represented severe dependency or mortality. For analytical purposes, outcomes were dichotomized into ‘favorable’ (GCS 13–15) and ‘unfavorable’ (GCS ≤ 12, or death). This binary threshold was specifically chosen to mirror the universally accepted dichotomy of the WFNS grading system (good-grade vs. poor-grade) [9].

### 2.4. Statistical Analysis

Numerical variables are summarized as mean ± standard deviation (SD), whereas categorical variables are expressed as absolute frequencies and percentages. Comparisons of categorical variables between groups were performed using Pearson’s chi-square test, or Fisher’s exact test when expected cell frequencies were small. Differences in continuous variables between two groups were assessed using the independent samples Student’s *t*-test. Bivariate analyses were conducted to explore associations between the presence and severity of Frank’s sign and relevant clinical and aneurysm morphological variables. Subsequently, multivariate logistic regression models were constructed to evaluate the independent association between high-grade DELC and aneurysm rupture, as well as rupture occurring at small aneurysm sizes (<7 mm). Multivariate logistic regression Model 1 evaluated overall aneurysm rupture adjusted for age, sex, maximum aneurysm size (mm), AComA/ACA location, hypertension, and smoking status. Model 2 evaluated rupture strictly within the subgroup of small aneurysms (<7 mm, n=117), adjusted for age, sex, AComA/ACA location, Aspect Ratio (≥1.6), hypertension, and smoking status. Because the DELC has been shown to correlate strongly with age-related vascular degeneration, age was included as a continuous covariate in all multivariate models to control for potential confounding [13]. A two-sided *p*-value < 0.05 was considered statistically significant. All analyses were performed using Stata statistical software version 14 (StataCorp, College Station, TX, USA).

## 3. Results

### 3.1. Study Population and Baseline Characteristics

A total of 186 consecutive patients with confirmed IA were included in the final analysis. The mean age of the study cohort was 58.5 years (SD ± 13.2), with a significant female predominance (74.7%, n = 139). Classical cardiovascular risk factors were prevalent among the patients, including hypertension (58.1%), dyslipidemia (32.3%), smoking (25.8%), and type 2 diabetes mellitus (21.0%). A favorable clinical outcome at discharge was observed in 56.5% of the cohort. The baseline demographic and clinical characteristics are summarized in Table 1.

### 3.2. Association Between DELC, Aneurysm Morphology and Rupture

**Figure 2:**
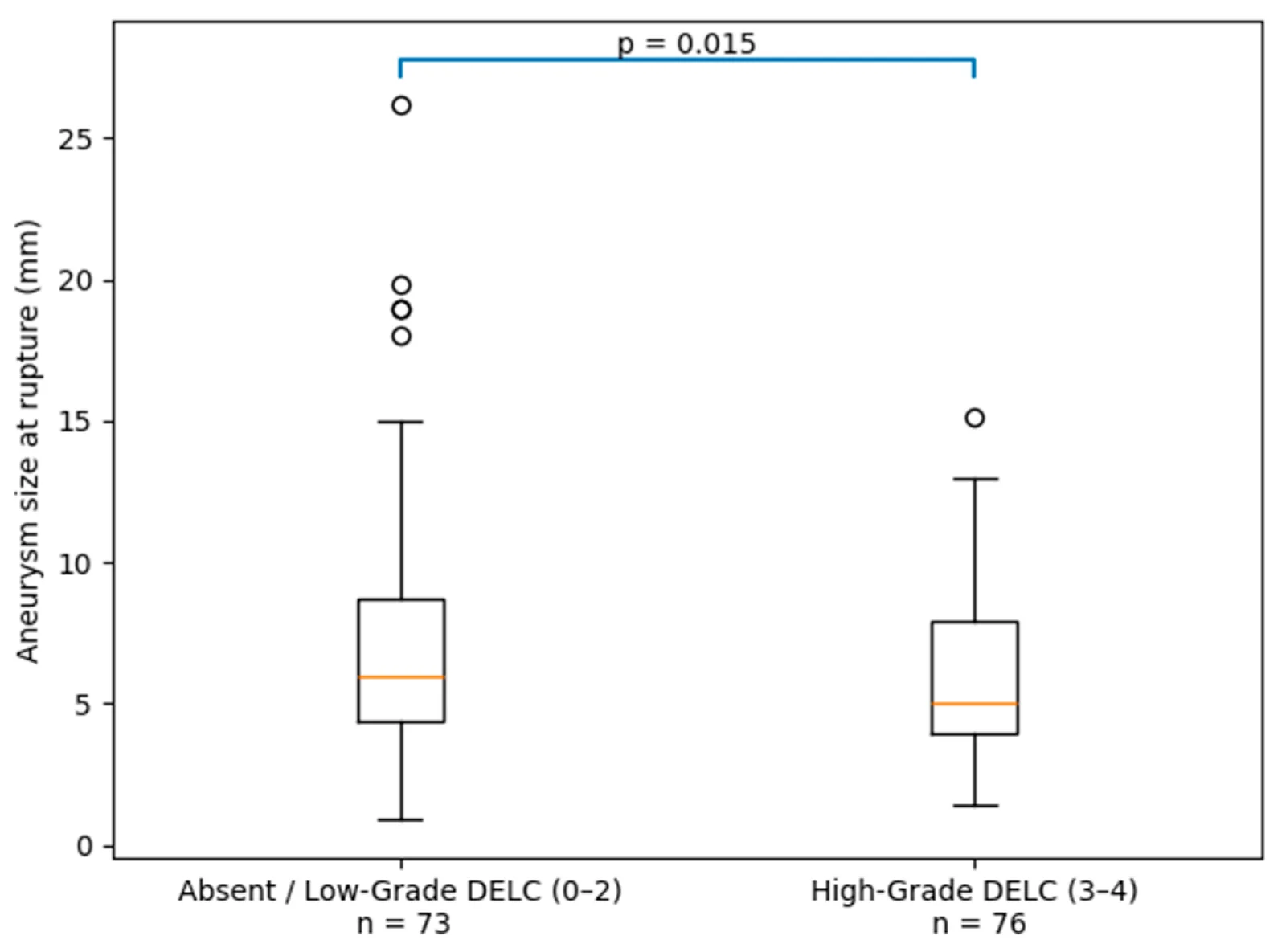
**Association between Frank’s sign severity and aneurysm size at rupture.** Box-and-whisker plot comparing the maximum aneurysm dome size at rupture among patients presenting with aSAH (n = 149). Patients were stratified according to the severity of the Frank’s sign into absent/low-grade DELC (grades 0–2; n = 73) and high-grade DELC (grades 3–4; n = 76). The central horizontal line indicates the median, the box bounds represent the interquartile range (IQR), whiskers extend to 1.5 × IQR, and individual circles represent outlying values beyond 1.5 × IQR. Patients with high-grade DELC experienced aneurysm rupture at significantly smaller sizes compared with those with absent or low-grade creases (*p* = 0.015). Notably, rupture frequently occurred below the conventional 7-mm risk threshold described in landmark natural history studies, supporting the concept that Frank’s sign may identify an additional marker of structural wall vulnerability before aneurysms reach conventionally high-risk dimensions.

Bivariate analysis revealed a statistically significant association between the presence of a high-grade DELC and aneurysm rupture. Specifically, a high-grade DELC was significantly more frequent in patients presenting with aSAH compared to those with unruptured aneurysms (*p* = 0.002). Furthermore, morphological assessment demonstrated that, among patients presenting with aSAH, those exhibiting a high-grade DELC experienced aneurysm rupture at significantly smaller mean sizes (6.11 mm ± 3.57) compared to those with absent or low-grade creases (7.94 mm ± 6.40, *p* = 0.015) (Table 2, Figure 2).

Regarding intrinsic vessel wall instability, a high-grade DELC was significantly associated with aneurysm rupture at small sizes (<7 mm) (*p* = 0.022). Additionally, significant differences were observed regarding anterior versus posterior circulation distribution (*p* = 0.024) and specific aneurysm location (*p* = 0.021) depending on the DELC grade. Notably, while the Posterior Communicating Artery (PComA) was the most frequent location in the absent/low-grade group, patients harboring a high-grade DELC exhibited a distinct anatomical distribution, with the Anterior Communicating Artery (AComA) becoming the predominant site of aneurysm formation (35.7%) (Table 2).

### 3.3. Multivariate Logistic Regression Analysis

To evaluate the independent statistical association of Frank’s sign, two multivariate logistic regression models were constructed (Table 3). In the first model, predicting overall aneurysm rupture adjusted for age, hypertension, and smoking status, a high-grade DELC demonstrated a strong independent association (Adjusted OR = 3.42, 95% CI: 1.39–8.41, *p* = 0.007). Notably, classical risk factors did not reach statistical significance in this model. This finding demonstrates that high-grade DELC is significantly associated with overall aneurysm rupture after adjusting for maximum aneurysm size, AComA/ACA location, and clinical covariates.

In the second model, restricted strictly to the subgroup of small aneurysms (<7 mm, n = 117), a high-grade DELC remained independently associated with rupture status (Adjusted OR = 2.08, 95% CI: 1.03–4.21, *p* = 0.041) after adjusting for AComA/ACA location (Adjusted OR = 2.34, 95% CI: 1.08–5.07, *p* = 0.031) and morphological covariates. In this model, an AR ≥ 1.6 demonstrated an inverse statistical association (Adjusted OR = 0.38, 95% CI: 0.18–0.79, *p* = 0.010).

Finally, to evaluate the impact of DELC on the clinical prognosis following aneurysmal rupture, a third multivariate logistic regression model was constructed strictly for patients presenting with aSAH (n = 149). The model was adjusted for established prognostic scales (WFNS and mFisher grades) and the type of neurosurgical procedure. In this analysis, clinical outcome at discharge was overwhelmingly dictated by admission neurological status (WFNS grade: OR = 27.07, *p* < 0.001) and radiological hemorrhage severity (mFisher grade: OR = 3.89, *p* = 0.012). As expected, once the structural failure of the vessel wall occurred, the macroscopic cutaneous marker (high-grade DELC) did not independently predict downstream neurological morbidity (*p* = 0.872), reinforcing its specific role as a marker of intrinsic wall instability rather than post-hemorrhagic clinical evolution.

## 4. Discussion

The present study suggests that DELC, or Frank’s sign, is not only associated with the presence of IA [7,8], but may also be independently associated with aneurysm rupture status. Our multivariate models demonstrated that high-grade DELC was associated with a nearly four-fold increase in the odds of overall aneurysm rupture and a two-fold increase in rupture among small aneurysms (<7 mm), which are conventionally managed conservatively. Notably, these associations remained statistically significant even after adjustment for classical cardiovascular covariates and strict geometric indices. Taken together, these findings support the hypothesis that Frank’s sign may represent an accessible clinical marker, an “intracranial window” associated with increased risk of aneurysm rupture and instability.

One of the key findings of our study is the association between high-grade DELC and aneurysm rupture at relatively small sizes. IAs pose a substantial global health burden, with aSAH causing severe morbidity and mortality [1,14]. Although preventive screening has been advocated, population-wide advanced neurovascular imaging remains logistically and economically unfeasible for the general population [2,15]. Recent evidence further suggests that the burden of aSAH disproportionately affects vulnerable populations with limited access to specialized neurovascular care [16]. In this context, the identification of a simple and readily observable clinical marker such as Frank’s sign may contribute to a more targeted use of advanced neuroimaging resources. Current management paradigms for unruptured intracranial aneurysms are heavily influenced by landmark studies such as ISUIA and UCAS Japan, which suggest that aneurysms smaller than 7 mm generally carry a low risk of rupture [11,12]. However, our data suggest that patients with high-grade DELC experience aneurysm rupture at significantly smaller mean sizes (6.11 mm) compared with those without the sign (7.94 mm). Our findings indicate that high-grade DELC is significantly associated with aneurysm rupture across the entire cohort. Notably, the association with rupture at small sizes (<7 mm) is particularly distinct, suggesting that in patients with high-grade DELC, systemic connective tissue degeneration may contribute additional biological information beyond conventional size-based risk assessment, an observation that warrants further clinical investigation.

Furthermore, in our second multivariate model (predicting rupture at small sizes), a high AR (≥1.6)—a well-established geometric index associated with high rupture risk [17,18]—showed an inverse association with rupture at small sizes (<7 mm). This geometric association suggests that while typical aneurysms may require a higher AR to rupture, aneurysms in patients with high-grade DELC may rupture before reaching conventionally high-risk morphologic features. This concept is further supported by the distinct anatomical distribution observed in our bivariate analysis (Table 2). Patients with a high-grade DELC exhibited a significantly higher proportion of aneurysms located at the AComA complex. Because the AComA complex represents one of the principal sites of hemodynamic shear stress within the Circle of Willis [19], a vessel wall potentially affected by systemic connective tissue remodeling (as marked by DELC) may be particularly susceptible to early aneurysm instability at this specific bifurcation [6,20,21]. Collectively, these findings suggest that patients with high-grade DELC may represent a subgroup with increased susceptibility to aneurysm instability, in whom rupture may occur before the development of conventionally high-risk morphologic features. Furthermore, the higher prevalence of AComA aneurysms observed in the high-grade DELC group requires careful interpretation. Because AComA aneurysms inherently tend to rupture at smaller dimensions due to distinct local hemodynamic shear stress, this anatomical distribution may act as a confounding factor in the relationship between DELC and small-size rupture. Additionally, the inverse association observed for Aspect Ratio (≥1.6, OR = 0.38) in Model 2 is best understood as a mathematical artifact resulting from the binary outcome thresholding (<7 mm) or collider stratification bias within a selected hospital cohort, rather than a protective biological effect.

Historically, the pathophysiological implications of DELC have been heavily centered on coronary artery disease since its first description by S.T. Frank in 1973 [3]. Systematic reviews and histopathological studies have consistently associated DELC with systemic atherosclerosis and cardiovascular burden [4,5]. However, emerging evidence suggests that DELC may reflect a broader pattern of systemic microvascular and connective tissue degeneration. Recent prospective studies and literature reviews have further associated Frank’s sign with cerebrovascular events, including ischemic stroke and transient ischemic attacks [22,23,24,25,26]. Crucially, this association remains statistically significant even after excluding patients with pre-existing cardiovascular disease [23,27], suggesting that DELC may be associated with an underlying state of systemic cerebrovascular vulnerability.

The transition from predicting ischemic events to predicting hemorrhagic neurovascular pathology may represent a plausible pathophysiological continuum. The histological hallmarks of Frank’s sign—including microvascular loss, aggressive collagen remodeling, and elastin degradation [4]—share important similarities with the physiological breakdown of the internal elastic lamina involved in intracranial aneurysm formation and instability [6,20]. Pathological analyses of ruptured aneurysms consistently demonstrate severe wall degradation, macrophage infiltration, and inflammatory remodeling [21,28,29]. Recent advances in high-resolution vessel wall imaging have further supported the association showing that atherosclerotic and inflammatory changes in the aneurysm wall correlate with aneurysm instability and increased rupture risk [30,31]. In this context, our clinical findings are consistent with this shared pathophysiological framework: the visible cutaneous changes observed in DELC may reflect systemic extracellular matrix remodeling processes that also contribute to aneurysm wall instability [8].

Furthermore, the potential clinical implications of this cutaneous marker may extend to the identification of otherwise unrecognized vascular vulnerability. Established risk factors for aSAH, such as smoking and hypertension, do not fully account for all aneurysm ruptures [32,33]. In our cohort, Frank’s sign was associated with rupture at smaller aneurysm sizes even among demographic subgroups traditionally considered at lower risk, including younger patients and those without a smoking history. Importantly, our third multivariate model demonstrated that while high-grade DELC was associated with aneurysm rupture status, it was not independently associated with downstream neurological morbidity, which remained primarily influenced by admission severity (WFNS and mFisher grades). These findings further support the hypothesis that DELC may be more closely related to pre-hemorrhagic aneurysm instability than to post-hemorrhagic neurological prognosis.

**Figure 3:**
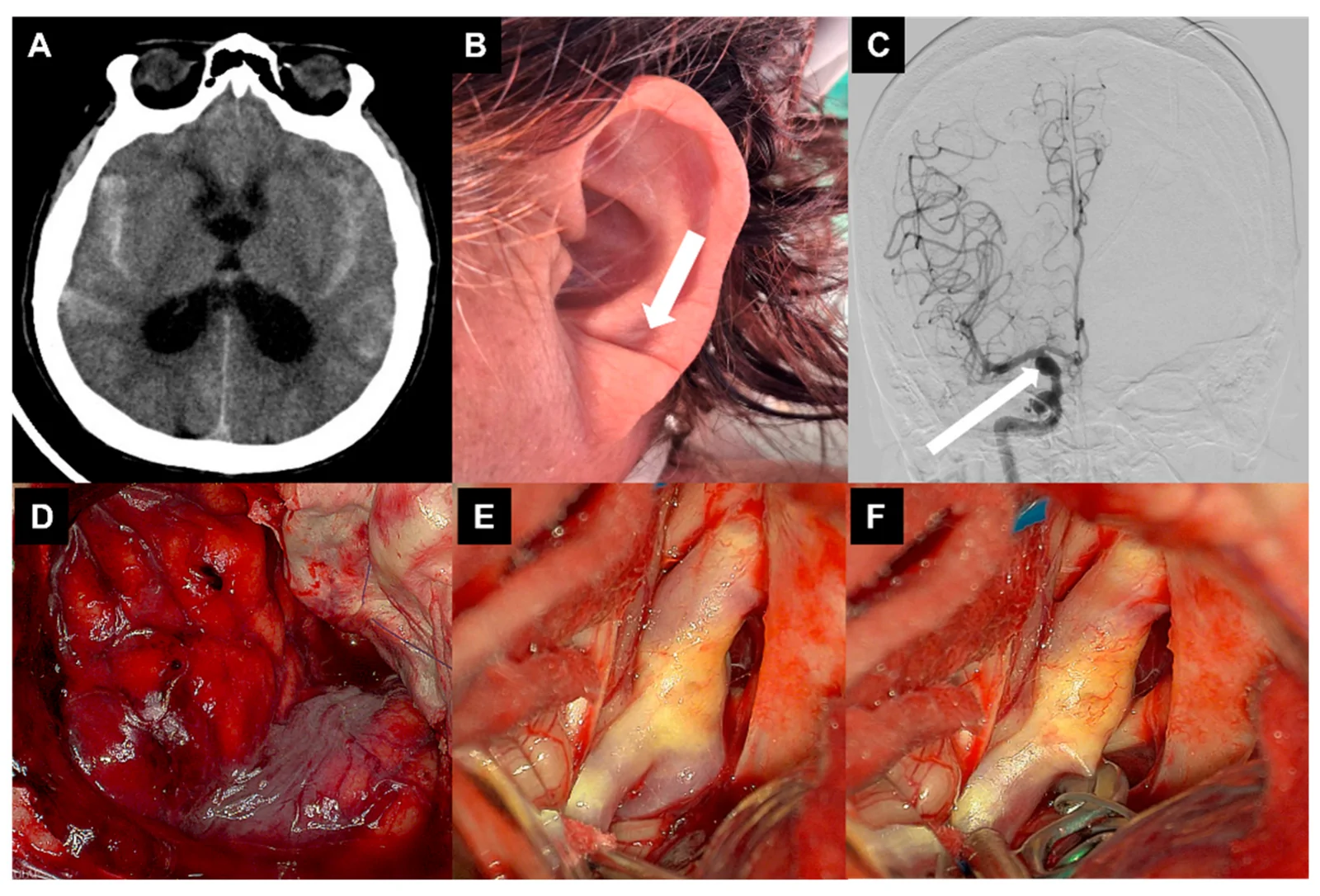
Illustrative case reflecting the clinical pathway leading to the hypothesis of potential cerebrovascular fragility associated with Frank’s sign. Comprehensive multi-panel representation of a patient presenting with aSAH from a small PComA aneurysm (<7 mm). (**A**) Non-contrast CT demonstrating diffuse subarachnoid hemorrhage predominantly involving the Sylvian cisterns and convexity subarachnoid spaces, with associated hydrocephalus. (**B**) Clinical photograph showing a deep and complete DELC (white arrow), consistent with high-grade DELC (Type 4). (**C**) Preoperative DSA identifying a small PComA aneurysm (<7 mm) indicated by the white arrow. (**D**) Intraoperative view after dural opening demonstrating extensive subarachnoid hemorrhage. (**E**) Intraoperative microphotograph during microsurgical dissection showing macroscopic wall changes of the supraclinoid Internal Carotid Artery (ICA), characterized by yellow atheromatous discoloration and areas suggestive of focal wall thinning proximal to the origin of the small PComA aneurysm. (**F**) Final intraoperative microsurgical view after aneurysm exclusion with clip reconstruction, where the abnormal appearance of the parent vessel wall remains visible adjacent to the clipped neck. This illustrative sequence reflects the recurrent clinical observation that motivated the present hypothesis: patients with high-grade DELC presenting with severe aSAH despite harboring relatively small aneurysms. In this context, Frank’s sign may be viewed as a potential “intracranial window”, linking an external marker associated with connective tissue remodeling to intraoperative observations of abnormal arterial wall appearance. These findings raise the possibility of a shared process of cerebrovascular vulnerability, warranting further investigation into the relationship between systemic tissue degeneration and aneurysm wall instability.

Overall, these findings suggest that Frank’s sign may serve as a visible extracranial surrogate reflecting a potential pattern of cerebrovascular fragility (Figure 3). While prospective mechanical and molecular validation is certainly required, this working hypothesis provides a reasonable clinical rationale linking macroscopic cutaneous changes with early aneurysm wall instability. In this context, Frank’s sign should not be interpreted as a definitive diagnostic tool, but rather as a simple, zero-cost clinical clue potentially associated with systemic connective tissue degeneration and aneurysm wall instability. This framework may help explain why certain aneurysms rupture despite conventionally low-risk morphologic features and small size (<7 mm). Importantly, these observations do not contradict the well-established paradigm that larger aneurysms inherently carry a higher absolute risk of rupture due to increased hemodynamic wall tension. Rather, our data suggest that in the presence of systemic connective tissue degeneration (high-grade DELC), intrinsic wall fragility may contribute beyond size-dependent biomechanical stress, potentially facilitating earlier aneurysm instability. Furthermore, our findings support the need for future multicenter studies integrating clinical phenotyping, high-resolution vascular imaging, and molecular characterization of the aneurysm wall. Ultimately, readily observable phenotypic markers such as DELC may contribute to more individualized approaches to aneurysm risk stratification and the early identification of aneurysm instability.

## 5. Limitations

This study has several limitations that must be acknowledged. First, as a single-center study conducted in a tertiary neurosurgical referral center, our cohort presents an inherent selection and spectrum bias, with a heavy weighting toward emergency ruptured cases (n = 149) compared to elective unruptured cases (n = 37), reflecting specialized hospital referral pathways rather than general population incidence. Second, the cross-sectional and observational design precludes establishing longitudinal causality or true prospective rupture prediction. Third, although models were adjusted for age, sex, hypertension, and smoking, residual confounding by unmeasured variables (such as specific genetic traits, systemic collagen alterations, or hemodynamic stress) cannot be fully excluded. Fourth, clinical outcome was evaluated using discharge GCS as a surrogate for acute neurological recovery rather than validated long-term functional disability scales such as the modified Rankin Scale (mRS) or Glasgow Outcome Scale (GOS). Finally, external validation in prospective, multicenter cohorts is necessary before these observational associations can be generalized.

## 6. Conclusions

Our findings suggest that high-grade DELC may represent a readily observable clinical marker associated with intracranial aneurysm instability and a higher susceptibility to rupture at small sizes (<7 mm). The observation of rupture occurring at small aneurysm sizes, often independently of conventionally high-risk morphologic features, supports further investigation into whether selected patients with high-grade DELC may harbor increased susceptibility to aneurysm rupture. Although our study was not designed to evaluate population-level screening, the simplicity and zero-cost nature of Frank’s sign support future exploration of this cutaneous marker as a potential adjunct for identifying individuals who may benefit from targeted neurovascular imaging. In the clinical setting, recognition of high-grade DELC may contribute to more individualized risk assessment in selected patients with small unruptured intracranial aneurysms.

## Figures and Tables

**Table 1:** Baseline Demographic and Clinical Characteristics and Aneurysm Location of the Cohort (*N* = 186).

Characteristic	Frequency (%)/Mean (SD)
Age, years (Mean, SD)	58.5 (13.2)
Sex	
Female	139 (74.7%)
Male	47 (25.3%)
Hypertension	108 (58.1%)
Dyslipidemia	60 (32.3%)
Smoking Status	48 (25.8%)
Diabetes Mellitus Type 2	39 (21.0%)
Aneurysm Circulation	
Anterior	164 (88.2%)
Posterior	22 (11.8%)
Aneurysm Anatomical Location	
PComA	62 (33.3%)
AComA/ACA	50 (26.9%)
MCA	41 (22.0%)
Other ICA Segments	11 (5.9%)
Posterior Circulation	22 (11.8%)
- Basilar Artery	10 (5.4%)
- PICA/Vertebral Artery	6 (3.2%)
- Superior Cerebellar Artery	4 (2.2%)
- Posterior Cerebral Artery	2 (1.1%)
Hospital Length of Stay, days (Mean, SD)	13.9 (8.9)
Clinical Outcome at Discharge	
Favorable	105 (56.5%)
Unfavorable	81 (43.5%)
Overall Mortality	26 (14.0%)

Abbreviations: PComA, Posterior Communicating Artery; AComA, Anterior Communicating Artery; ACA, Anterior Cerebral Artery; MCA, Middle Cerebral Artery; ICA, Internal Carotid Artery. PICA, Posterior Inferior Cerebellar Artery.

**Table 2:** Bivariate Analysis of Aneurysm Characteristics according to Frank’s sign Grade.

Characteristic	Absent/Low-Grade DELC (n = 102)	High-Grade DELC (n = 84)	*p* Value
Aneurysm Rupture			0.002 *
Unruptured	29 (28.4%)	8 (9.5%)	
Ruptured	73 (71.6%)	76 (90.5%)	
Dichotomized Aneurysm Size			0.264
<7 mm	60 (58.8%)	57 (67.9%)	
≥7 mm	42 (41.2%)	27 (32.1%)	
Rupture at Small Size (<7 mm)			0.022 *
No	56 (54.9%)	32 (38.1%)	
Yes	46 (45.1%)	52 (61.9%)	
Maximum Aneurysm Size at Rupture, mm (Mean, SD) ^†^	7.94 (6.40)	6.11 (3.57)	0.015 *
Circulation			0.024 *
Anterior	85 (83.3%)	79 (94.0%)	
Posterior	17 (16.7%)	5 (6.0%)	
Aneurysm Anatomical Location			0.021 *
PComA	39 (38.2%)	23 (27.4%)	
AComA/ACA	20 (19.6%)	30 (35.7%)	
MCA	19 (18.6%)	22 (26.2%)	
Posterior Circulation	17 (16.7%)	5 (6.0%)	
Other ICA Segments	7 (6.9%)	4 (4.8%)	

^†^ Calculated exclusively for the subgroup of patients presenting with aneurysmal rupture (Absent/Low-Grade DELC, n = 73; High-Grade DELC, n = 76). Abbreviations: PComA, Posterior Communicating Artery; AComA, Anterior Communicating Artery; ACA, Anterior Cerebral Artery; MCA, Middle Cerebral Artery; ICA, Internal Carotid Artery. *: Statistically significant at *p* < 0.05.

**Table 3:** Multivariate Logistic Regression Models Predicting Aneurysm Rupture, Rupture at Small Sizes (<7 mm), and Clinical Outcome.

Predictor Variable	Adjusted Odds Ratio (OR)	95% Confidence Interval	*p* Value
Model 1: Overall Aneurysm Rupture			
High-Grade DELC	3.42	1.39–8.41	0.007
Maximum Size (mm)	1.12	1.02–1.23	0.018
AComA/ACA Location	2.15	1.01–4.58	0.047
Age	1.00	0.97–1.03	0.912
Sex	1.21	0.51–2.86	0.662
Hypertension	1.10	0.48–2.50	0.821
Smoking Status	1.05	0.42–2.60	0.918
Model 2: Rupture in Small Aneurysms Subgroup (<7 mm) (n = 117)			
High-Grade DELC	2.08	1.03–4.21	0.041
AComA/ACA Location	2.34	1.08–5.07	0.031
Age	1.01	0.98–1.04	0.612
Sex	0.92	0.38–2.21	0.851
Hypertension	1.04	0.49–2.22	0.918
Smoking Status	0.91	0.41–2.02	0.819
Aspect Ratio (AR) ≥ 1.6	0.38	0.18–0.79	0.001
Model 3: Predicting Unfavorable Clinical Outcome (Ruptured cohort only, n = 149)			
High-Grade DELC	0.93	0.41–2.12	0.872
WFNS Grade (Poor)	27.07	7.78–94.12	<0.001
modified Fisher Grade (High)	3.89	1.35–11.22	0.012
Surgical or endovascular treatment	3.72	0.70–19.58	0.121

(Note: Rupture at small sizes in Model 2 is defined as aneurysm rupture occurring at a maximum dome size of <7 mm).

## Data Availability

The data presented in this study are available on request from the corresponding author due to privacy and ethical restrictions regarding clinical patient records.
